# An Integrated Photoluminescence Sensing Platform Using a Single-Multi-Mode Fiber Coupler-Based Probe

**DOI:** 10.3390/s140305677

**Published:** 2014-03-21

**Authors:** Feng Long, Anna Zhu, Hanchang Shi

**Affiliations:** 1 School of Environment and Natural Resources, Renmin University of China, No.59, Zhongguancun, Haidian, Beijing 100872, China; 2 Department of Chemistry, Massachusetts Institute of Technology, 77 Massachusetts Ave, Cambridge, MA 02139, USA; E-Mail: Zhuanna00@mails.thu.edu.cn; 3 School of Environment, Tsinghua University, No.1, Tsinghua Yuan, Haidian, Beijing 100084, China

**Keywords:** photoluminescence, optical fiber coupler, fiber probe, fluorescence measurement

## Abstract

We demonstrate an integrated fiber optic photoluminescence sensing platform using a novel single-multi-mode fiber coupler (SMFC)-based probe with high collection efficiency for fluorescence signals. The SMFC, prepared using fused biconical taper technology, not only transmits excitation light, but also collects and transmits fluorescence. The entire system does not use complex optical components and rarely requires optical alignment. The simple structure of the SMFC considerably improves the light transmission efficiency, signal-to-noise ratio, and sensitivity of the system. Theoretical and experimental results show that the proposed probe increases the collection efficiency by more than eight-fold compared with a bifurcated fiber probe. The performance of the proposed probe was experimentally evaluated by measuring the fluorescence spectra of well-known targets and a fresh Tall Fescue leaf.

## 1. Introduction

Photoluminescence (PL) measurement systems have become an invaluable tool for noninvasive and *in vitro*/*in vivo* detection; such systems have been extensively applied in biomedical, biological, chemical, and environmental assays [1–3]. The conventional PL measurement system typically incorporates a light source, a spectrometer, and an optical transmission module including a probe, enabling it to deliver excitation and collection light. The optical transmission structure has many optical components, such as an off-axis parabolic reflector, dichroic beam splitters, and a biconvex silica lens. This bulk optical arrangement is high cost and requires crucial optical alignment. Once the direction of any element becomes inaccurate, the whole system will be useless and be very difficult to reconfigure. The design of the probe is critical to improve the sensitivity of a PL measurement system [2]. Optic fiber probes are often used because of their flexibility, cost-effectiveness, compactness, and remote monitoring capability [2,3].

Various optical fiber probes, such as single-fiber [4], bifurcated [5,6], and coaxial fiber probes [7], have been incorporated into PL measurement systems. A single-fiber probe, which delivers light to a sample, is also used for collection. It exhibits excellent light collection efficiency because the overlap between the excitation light and collection region is complete and independent of the distance from the probe tip [4]. However, the coupling of excitation light and the collection and detection of fluorescence require complex optical components and rigid optical alignment for conventional single-fiber probes. Moreover, the returning emitted/scattered light needs to be separated from the excitation light with optical devices, such as an optical circulator or a dichroic beam splitter. The use of such devices can result in interference problems and reduce signal-to-noise (S/N) ratios. Bifurcated probes or coaxial fiber probes do not present problems related to fluorescence background signals because they only use one fiber to deliver excitation light and another fiber to collect signals from samples. Nevertheless, these probes have low fluorescence collection efficiency because they offer poor spatial overlapping between the cone in which the excitation light propagates and the fluorescence collection cone.

Researchers have attempted to improve the efficiency of fluorescence collection by using the double-clad fiber probe [8], which was constructed from a single-fiber piece, but operated as a multi-fiber probe. However, this design requires a special fiber coupler because the fluorescence signals collected from samples cannot be directly delivered to the detector. Moreover, the reported coupling efficiency of the special fiber coupler is less than 40%. Lens-coupled optical fiber probes for fluorescence measurements were also introduced [9]. These probes present advantages, such as compact size, short working distance, and effective collection of localized fluorescence signals. However, the fabrication of optical assembly for these configurations is relatively complex and requires precise alignment of optical components.

In this study, we demonstrate a simple and compact PL sensing platform using a special single-multi-mode fiber coupler (SMFC)-based probe for effective collection of fluorescence signals. The SMFC enables the transmission of excitation light and the collection and transmission of fluorescence. The proposed PL system has many benefits, such as simple fabrication, compactness, excellent light collection efficiency, minimal requirement of optical components, and rare need for optical alignment.

## 2. System Configuration

**Figure 1.**
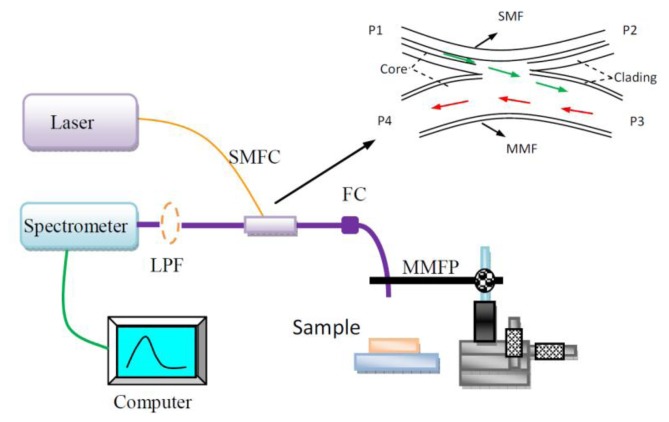
Schematic of the photofluorescence sensing platform based on the proposed optical probe: SMFC, single-multi-mode fiber coupler; FC, fiber connection; LPF, long pass filter; MMF, multi-mode fiber; SMF, single-mode fiber; Inset: schematic of the SMFC coupler. The laser light coming along the core of the P1 port is mostly delivered into the core of the P3 port; the fluorescence collected by the P3 port was directly coupled to the P4 port.

Figure 1 shows the schematic of a PL sensing platform that uses the proposed SMFC-based all-fiber probe. Instead of using lenses and dichroic mirrors, this coupler transmits excitation light and collects and transmits fluorescence. The SMFC was prepared by fused biconical taper technology as previously described [10]. Its structure is shown in the inset of Figure 1. A 9 μm/125 μm (core/cladding diameters) single-mode fiber and a 580 μm/610 μm multi-mode fiber (NA = 0.22) were removed from the plastic cladding and placed in contact with each other. The contact region was heated and fused; meanwhile, the assembly was elongated and tapered. The input and out ends are identical; thus, either side may be used as the input end. Assuming that the power (*P*_in_) injected into the P1 port is unity, the power (*P*_out_) in the P3 port evolves as [3]:

(1) Pout=Pinsin(Δ∅2)2

where ΔΦ is the cumulative phase difference between the even and odd supermodes, and is given by:

(2) $$\Delta \emptyset =\int_{-\text{L}}^{+\text{L}}\left(\beta_{\text{e}}-\beta_{0}\right)\text{dz}$$

where *β*_e_ is the propagation constant of the even supermode, *β*_0_ is the phase constant of the odd mode, *L* represents the extent of taper on either side of the center of the taper.

Considering that the fused couplers involve an axially varying complex waveguiding geometry consisting of cores, cladding, and the surrounding air, a rigorous mathematical analysis of such a device is a difficult task [11]. In our system, a 635 nm laser beam with a power of 5 mW was transmitted into the input port P1. During the “fusion and elongation” process, the output port P3 was monitored. The entire beam coming from the P1 port along the core of the single-mode fiber can be coupled to the multi-mode fiber and the output port P3 with the help of the proposed fiber coupler. When the coupling efficiency from P1 to P3 reached more than 80%, elongation was halted. The same measurement was repeated from P3 to P4, generating a coupling efficiency of more than 80%. The coupling efficiency from P3 to P1 was almost 0 because the core diameter of the single-mode fiber was only 9 μm. P2 had no function in this experiment and was therefore blocked.

The excitation light from a fiber pigtailed laser diode (635 nm central wavelength, 5 mW; BWT Beijing, China) was launched into the single-mode fiber of the SMFC. The single-mode fiber approximately 9 μm in diameter only transmits one mode light. Thus, the light in it will rarely be lost because of the twist in use. Then, the laser light was delivered to the multi-mode fiber (NA = 0.22, id = 600 μm) of the SMFC through the single-mode to multi-mode coupling structure. A 4.1 mW output was obtained from the distal end of the multi-mode fiber. A broadband spectrometer (QE65000, Ocean Optics, Dunedin, FL, USA) with the spectral range of 300 to 1,050 nm was used to analyze the PL signals through the multi-mode fiber of the SMFC, which was interrogated by a laptop via USB connection. A high pass filter at 650 nm was employed at the detector to block the fundamental excitation light that was elastically reflected and scattered back from the sample. The Ocean Optics SpectraSuite software package was used to record the fluorescence spectra. The system is suitable for *in situ* or *in vitro*/*in vivo* detection in biomedical, chemical, and environmental engineering because its structure is simple and does not require complex optical components and rigid optical alignment.

## 3. Theoretical Comparison of Bifurcated Probe and Single Multi-Mode Fiber Probe

**Figure 2.**
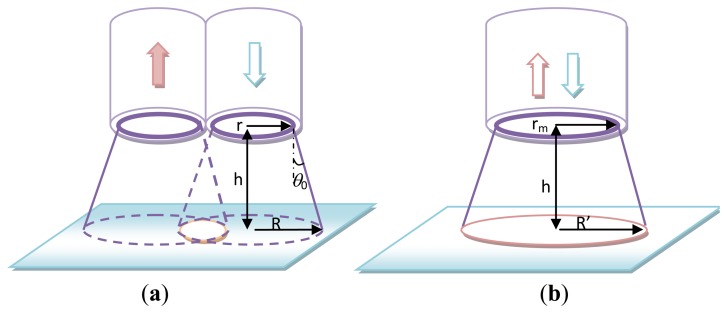
(**a**) Typical bifurcated optical fiber probe; (**b**) Single multi-mode fiber probe for enhanced light collection.

Previous optical fiber PL sensing systems required two optical paths, in which one optical fiber probe was used for excitation laser delivery and the other was used for PL signal collection [11,12]. In these systems, the PL signal was collected by an additional bulk microscopic objective lens system [11]. In the present study, we constructed a bifurcated probe (Figure 2a) to replace the SMFC in the same photofluorescence sensing platform for performance comparison purposes. The excitation light and the captured signal light for a bifurcated probe are limited by the excitation cone and the receiving cone represented by 2*θ*_0_, respectively. In the most common construction, which has two identical fibers, the angles of these two cones that open to the sample are equal and determined by their numerical aperture NA = *n* sin*θ*_0_, where *n* is the refractive index of the air. The captured fluorescent light from the sample can only arise from this overlapping or active area.

Assuming that the distance between the end surface of the bifurcated probe and the fluorescence detection area is *h*, we can calculate the overlapping area by [3]:

(3) $$\text{S}\left(\text{h}\right)=2\cdot \left[{\text{R}}^{2}\cdot \sec^{-1}\left(\frac{\text{R}}{\text{r}}\right)-\text{r}\sqrt{{\text{R}}^{2}-{\text{r}}^{2}}\right]$$

where *r* and *R* are the radii of the fiber core and cross section at the height *h*, respectively. *R* can be written as:

(4) $$\text{R}=\text{h}\tan\theta_{0}+\text{r}$$

To simplify the discussion, we ignored the attenuation of the excitation light when traveling. The excitation power density *P_h_* at the height *h* can be calculated by:

(5) $${\text{P}}_{\text{h}}=\frac{{\text{P}}_{0}\cdot {\text{S}}_{0}}{{\text{S}}_{\text{h}}}$$

where *P*_0_ is the excited power density at the output of fiber probe. Given that *S*_0_ = πr^2^ and *S*_h_ = πR^2^, then:

(6) $${\text{P}}_{\text{h}}={\text{P}}_{0}\left(\frac{{\text{r}}^{2}}{{\text{R}}^{2}}\right)$$

The fluorescent intensity *I*_b_ related to the excited light intensity *I*_e_, molar absorptivity *ε*, quantum yield *Q*, and concentration *c* can be expressed as:

(7) $${\text{I}}_{\text{b}}=\text{k}\cdot \text{Q}\cdot {\text{I}}_{\text{e}}\left(h\right)\cdot ɛ\cdot \text{c}$$

where k is a constant coefficient that can be determined by calibration.

By applying Equation (7) to Figure 2a, we can obtain the total fluorescence intensity captured in the overlapping area as:

(8) $${\text{I}}_{\text{b}}=\text{k}\cdot \text{Q}\cdot {\text{I}}_{\text{e}}(\text{h})\cdot ɛ\cdot \text{c}$$

where:

(9) $${\text{I}}_{\text{e}}\left(\text{h}\right)={\text{P}}_{\text{h}}\cdot \text{S}\left(\text{h}\right)$$

Replacing the associated terms in Equations (8) with Equations (3) and (9) yields:

(10) $${\text{I}}_{\text{b}}=\gamma \cdot \left[{\text{R}}^{2}\cdot \sec^{-1}\left(\frac{\text{R}}{\text{r}}\right)-\text{r}\sqrt{{\text{R}}^{2}-{\text{r}}^{2}}\right]\cdot \left(\frac{{\text{r}}^{2}}{{\text{R}}^{2}}\right)$$

where γ = 2×k Q ·ε · c · P_0_.

For the single multi-mode fiber probe, the overlap between the excitation light and collection region is complete and independent of the distance from the probe. The total fluorescence intensity captured can be calcaluted by:

(11) $${\text{I}}_{\text{m}}=\gamma^{'}\cdot {\text{r}}_{\text{m}}^{2}$$

where γ’ = π · k · Q ·ε · c · P_0_., *r*_m_ is the radius of the multi-mode fiber probe.

Using both probes to detect the same material, we can express the ratio of the total fluorescence intensity detected by the bifurcated probe to that detected by the presented probe as:

(12) $$\rho =\frac{{\text{I}}_{\text{b}}}{{\text{I}}_{\text{m}}}$$

Based on Equations (10) and (12), we observed that the detected fluorescence signals were dependent on the distance *h*, inner diameter *r* of the fiber core, excited power intensity *I*_e_, molar absorptivity *ε*, quantum yield *Q*, and concentration *c*. For comparison, the same excited power intensity *I_e_* and the same fluorescence materials were used. Meanwhile, the following values were used as common parameters: *n*_core_ = 1.456, *n*_air_ = 1.000, laser source wavelength = 635 nm, and multi-mode fiber NA = 0.22. In our system, the inner diameter of the multi-mode fiber probe was 600 μm.

**Figure 3.**
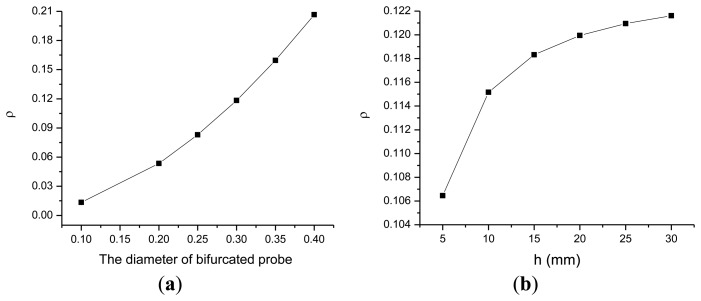
(**a**) Effect of the diameter of bifurcated probe on the fluorescence intensity ratio *ρ*. The distance between bifurcated/presented probe and sample is the same; (**b**) Effect of the distance between the end surface of the bifurcated probe and the fluorescence detection area on the fluorescence intensity ratio *ρ*.

Based on Equation (12), we evaluated the PL intensity ratio *ρ* between the bifurcated probe and the presented probe using different diameters of the fiber core of the bifurcated probe and different distance *h* values to investigate the effect of the distance *h* and the inner diameter *r* of the fiber core on the performance of both probes. The results of these calculations are shown in Figure 3. As shown in Figure 3a, *ρ* rapidly increases as the radius of the bifurcated probe increases. That is, the overlapping or active area linearly increases as the radius of the bifurcated probe increases. However, the PL intensity detected by the bifurcated probe is approximately 20% that of the single multi-mode optical fiber probe, even when the diameter of bifurcated probe is 400 μm. We used the 300 μm in diameter bifurcated probe for later experiments.

For the bifurcated probe consisting of two fiber probes 300 μm in diameter, *ρ* rapidly increases with distance between the probe and detection area when the distance is less than 15 mm (Figure 3b). Beyond this height, this ratio slowly increases and eventually reaches a plateau. Moreover, the ratio is less than 0.122. Therefore, the PL intensity detected by the bifurcated probe is approximately 12.2% that detected by the single multi-mode optical fiber probe. A coaxial fiber probe consisting of a laser fiber and six fluorescence collection fibers are generally used to improve the collection efficiency of fluorescence [6]. Given that six identical active areas are available for a coaxial optical fiber probe, the overall captured fluorescent power is increased by six fold. Therefore, the detection capacity of the coaxial optical fiber array for fluorescence signals is six times stronger than that of the bifurcated probe. However, the detection capacity of the coaxial optical fiber array remains lower than that of the single multi-mode fiber probe. Moreover, our proposed all-fiber device can provide optical paths in a single fiber unit for both the excitation laser and the PL signals, thereby significantly improving system integration capability and fluorescence collection efficiency.

## 4. Experiments

**Figure 4.**
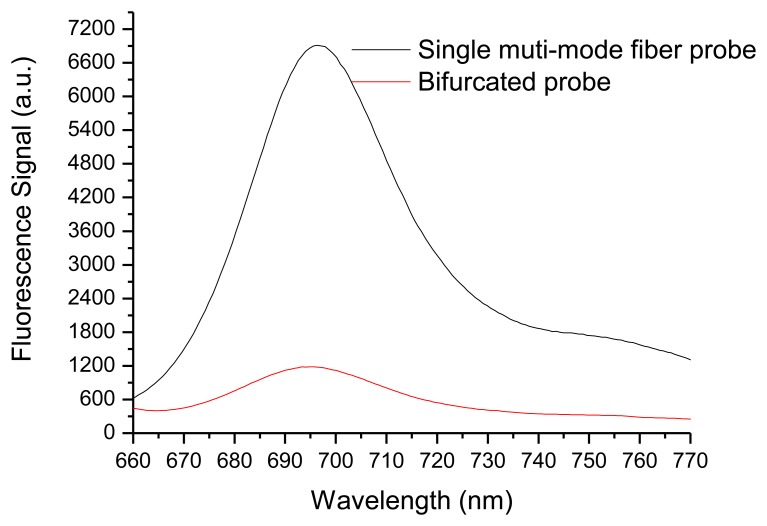
Fluorescence spectra of Cy5.5 agarose film with the single multi-mode fiber probe and bifurcated probe, respectively. The fluorescence spectra were measured at 200 μW and at an acquisition time of 100 ms.

Experiments were performed with a fluorescence dye-Cy5.5 agarose film and a fresh Tall Fescue leaf to verify the performance of the fabricated probe. The dye-Cy5.5 agarose film was prepared as follows: Cy5.5 solution was prepared by dissolving 0.5 μg Cy5.5 in a minimum amount of *N*,*N*-dimethylformamide and further diluting in 10 mL of 0.1 M phosphate-buffered saline as storage solution. A small amount of agarose solution was mixed with 10 nM Cy5.5 solution, and then the solution was poured into a PVC mold (cylindrical, 12 mm diameter; thickness, 300 μm). A thin microfilm was placed on top of the mold to ensure a flat surface, and the Cy5.5–agarose solution was solidified to form fluorescence film. The multi-mode fiber probe presented and the bifurcated probe were used to detect Cy5.5 film. The results are shown in Figure 4. All measurements in Figure 4 were taken at the same distance (15 mm). The fluorescence signal of the multi-mode fiber probe is approximately eight times higher than that of the bifurcated probe, which is similar to the results of theoretical analysis. This condition, which contributed primarily to the intensity and effective collection depth of the detected fluorescence signals, was determined by the overlap between the excitation light and collection region. For the bifurcated probe and coaxial optical fiber array, the overlap between the excitation light and fluorescence collection region is very small. However, for our multi-mode fiber probe, the overlap between the excitation light and fluorescence collection region is complete, indicating higher efficiency of fluorescence collection. Meanwhile, unlike the conventional single-fiber probe, the SMFC in this system provides the transmission of excitation light and the collection and transmission of fluorescence to eliminate the end-surface reflection of the conventional single-fiber probe because it uses a lens for focusing and a dichroic beam splitter to separate returning emitted/scattered light from excitation light. The simple and robust optical structure of our system has a higher S/N ratio. It is also suitable for *in situ* detection of real samples.

**Figure 5.**
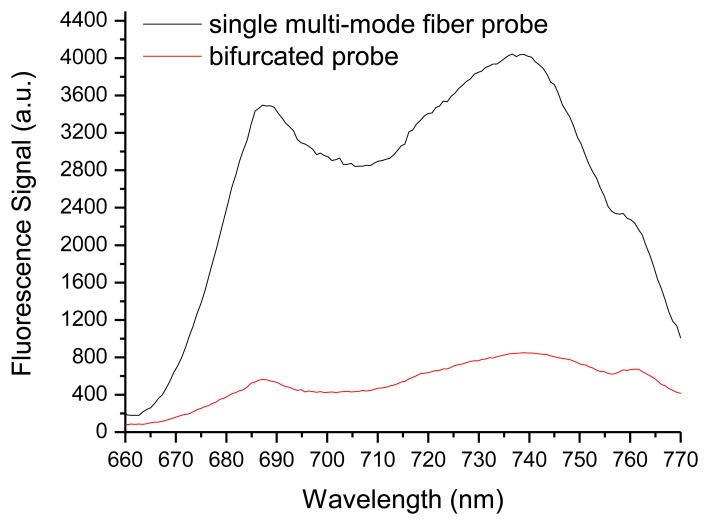
Fluorescence spectra of the Tall Fescue leaf. The fluorescence spectra were measured at 200 μW and at acquisition times of 200 ms for both the multi-mode fiber probe and bifurcated probe.

We also investigated the performance of the proposed probe to detect the fluorescence of real solid targets. Figure 5 shows the fluorescence spectra of a fresh Tall Fescue leaf, as measured at optimal working distances for both the multi-mode fiber probe and the bifurcated probe. For comparison, the excitation beam from a 635 nm laser diode was adjusted to have the same power of 200 μW at the sample arm, and the spectrum was taken at an acquisition time of 200 ms for the multi-mode fiber probe and the bifurcated probe. At an acquisition time of 200 ms, the fluorescence peak in the result of the bifurcated probe is unclear because of the low collection power. During the experiments, the dark current noise of the detector was subtracted before the spectral measurements were taken. Fluorescence emissions can be located at the two peak positions of the red and far-red wavelength regions. Figure 5 shows the two main peaks located at wavelengths of approximately 685 and 740 nm from the spectrum obtained with the proposed probe. The proposed multi-mode fiber probe collects eight times higher fluorescence signals than the bifurcated probe. This result demonstrates that the fluorescence detection system presented in this study can potentially be developed into a chlorophyll fluorometer with high sensitivity.

## 5. Conclusions

We have fabricated an integrated all-fiber PL sensing platform using a novel single multi-mode fiber coupler based probe. Its effectiveness in collecting fluorescence signals was theoretically and experimentally validated. The presented fiber probe effectively collects fluorescence signals with an eight-fold increase in collection efficiency compared with that of a bifurcated probe for the Cy5.5 film and a Tall Fesue leaf. The probe can be used to directly detect the chlorophyll fluorescence of grass leaves with high sensitivity. Therefore, the system presents promising potential for development into a chlorophyll fluorometer.

The main advantages of the proposed probe are as follows: (i) The SMFC for the transmission of excitation light, as well as the collection and transmission of fluorescence endow the system with high efficiency in fluorescence collection and a high S/N ratio, thereby significantly improving the system's sensitivity; (ii) The entire structure of the system is very simple and does not require complex optical components and rigid optical alignment, making it suitable for *in situ* or *in vitro*/*in vivo* detection in biomedical, chemical, and environmental engineering.
